# Engaging with Transformative Paradigms in Mental Health

**DOI:** 10.3390/ijerph18189504

**Published:** 2021-09-09

**Authors:** Louise Whitaker, Fiona L. Smith, Catherine Brasier, Melissa Petrakis, Lisa Brophy

**Affiliations:** 1 Faculty of Health, Southern Cross University, Coolangatta, QLD 4225, Australia; 2 Department of Occupational Therapy, Social Work and Social Policy, School of Allied Health, Human Services and Sport, La Trobe University, Bundoora, VIC 3083, Australia; f.smith@latrobe.edu.au (F.L.S.); catherine.brasier@latrobe.edu.au (C.B.); l.brophy@latrobe.edu.au (L.B.); 3 Department of Social Work, School of Primary & Allied Health Care, Monash University, Caulfield East, VIC 3145, Australia; melissa.petrakis@monash.edu; 4 Mental Health Service, St. Vincent’s Hospital Melbourne, Fitzroy, VIC 3065, Australia; 5 Centre for Mental Health, Melbourne School of Population and Global Health, University of Melbourne, Parkville, VIC 3010, Australia

**Keywords:** mental health, social work education, transformative education, professional education, critical practice, mental health social work, pedagogy

## Abstract

When graduates of Australian social work courses embark on a career in mental health, the systems they enter are complex, fragmented and evolving. Emerging practitioners will commonly be confronted by the loneliness, social exclusion, poverty and prejudice experienced by people living with mental distress; however, social work practice may not be focused on these factors. Instead, in accordance with the dominant biomedical perspective, symptom and risk management may predominate. Frustration with the limitations evident in this approach has seen the United Nations call for the transformation of mental health service delivery. Recognising paradigmatic influences on mental health social work may lead to a more considered enactment of person centred, recovery and rights-based approaches. This paper compares and contrasts influences of neo-liberalism, critical theory, human rights and post-structuralism on mental health social work practice. In preparing social work practitioners to recognise the influence of, and work more creatively with, intersecting paradigms, social work educators strive to foster a transformative approach to mental health practice that straddles discourses.

## 1. Introduction

Poor mental health outcomes globally continue to be unacceptable and, in this context, the United Nations Human Rights Council has called for a transformative paradigm because:

… the field of mental health continues to be over-medicalised and the reductionist biomedical model, with support from psychiatry and the pharmaceutical industry, dominates clinical practice, policy, research agendas, medical education and investment in mental health around the world. [1] (pp. 5–6).

In response, the United Nations Special Rapporteur has recommended a shift to a rights-based approach that directly addresses the power imbalance in mental health policies and services [2] due to concerns about colonising practices and the political nature of mental health care [3,4,5,6].

Social work, and in particular critical mental health social work, concurs. Social work is:

a practice profession and an academic discipline that recognises that interconnected historical, socio-economic, cultural, spatial, political and personal factors serve as opportunities and/or barriers to human wellbeing and development [7].

A recent review of the strategic role of social work in mental health in Northern Ireland, recognised “social work can bring a counterbalance to more individualised, more clinical and more treatment focused perspectives on mental health” [8] (p. 16). This captures the essence of social work values and ethics and is core to social work practice. However, in Australia, where mental health social work spans private practice, public healthcare and the non-government sector [9,10,11,12] these approaches are often “smuggled in” or subversive [13] within heavily prescribed roles. 

The recently revised Australian Social Work Education and Accreditation Standards (ASWEAS), with which our courses comply, recognise the multiple social and political factors influencing mental wellbeing [14]. The new standards position educators to address questions of power and injustice in mental health social work. However, the learning resources that support the teaching of paradigmatic influences on mental health social work are limited. Detailed exploration of these influences is rare in the mental health social work textbooks to which we refer [10,12,15,16,17,18,19,20,21]. Thus, in considering a social work contribution to transformations in mental health thinking and practice we began by exploring a selection of existing paradigms and their influence on mental health social work [22].

## 2. Methods

As mental health social workers who teach in this field, and consistent with the mandate of the profession, the authors of this paper came together to explore the place of social work education in preparing future practitioners in fully realising the profession’s potential to be engaged in system and practice transformation [23,24,25,26]. The authors met regularly over 8 months from November 2020 to draw on our research, pedagogy and practice wisdom and relevant literature to collectively critically reflect on discourses that shape mental health social work [27]. While some authors led sections, all of the authors collaborated on the conceptualisation, development, construction and final editing of the paper. Co-construction and co-writing fostered the rigor and trustworthiness of the paper [28]. 

The author’s pedagogy is influenced by their experience of co-production [29] as well as working within the dominant paradigm and critical feminist, human rights and post-structuralist approaches to mental health social work. 

## 3. Results

By integrating recognition of paradigmatic influences with reflective processes [30] we aim to reduce students’ conscription to essentialism and dualities [23,24,25,31]. Social work education requires:

the development of critical consciousness through reflecting on structural sources of oppression and/or privilege, on the basis of criteria such as race, class, language, religion, gender, disability, culture and sexual orientation, and developing action strategies towards addressing structural and personal barriers are central to emancipatory practice where the goals are the empowerment and liberation of people [7].

Turning this into pedagogy invites students to recognise the unique perspectives of multiple paradigms and the intersections, development and dominance, or otherwise, of their influence on mental health practice [27]. Ideally, we hope students gain literacy in crucial lines of thought, communication skills and reflective approaches that equip them for nuanced and targeted diplomacy. The act, or even art, of holding competing worldviews [30] equips students to work with multiple perspectives inclusive of service users, family and interdisciplinary team members. The intent is to enable the marriage of espoused theory and practice, promoting consistency and congruence [26]. It is envisaged that students will be prepared to recognise opportunities for championing transformative approaches to mental health service delivery [22,24] and, in turn, support the revisiting and revitalisation of the profession’s more radical contributions in mental health social work.

Our reflections highlighted influences of four well established social science discourses: neo-liberalism including the biomedical approach, critical theory, human rights, and post-structuralism. Our discussions acknowledged the challenges and opportunities each offers mental health social work, the people we serve and support, and the services in which we work.

### 3.1. Neo-Liberalism and Social Work in Mental Health

Neo-liberalism dominates contemporary mental health service delivery [32,33]. Neo-liberalism views people as self-concerned, producing and consuming individuals with needs, including those associated with mental wellbeing, best met through family or the marketplace. Building on the work of Foucault, Garrett [34] explores the state’s role, which is to engage in actively promoting the market economy where “economic processes and institutional frameworks … shape each other in ceaseless reciprocity” (p. 54). Humans become “capital” to be invested in, and within this “competitive enterprise” [34] the work of Foucault (p. 54) can be reconfigured and intensified for greater economic return [35]. In brief, viewed through this lens, mental health is understood in terms of individual functioning with indicators of “good practice” being value for money and minimal, yet effective, targeting of the state.

Conceptualisation of mental illness as a disease of the brain with physiological manifestations, that is, a biomedical view, aligns well with the individualistic focus of neo-liberalism. Despite the dangers of such a reductionist perspective, the insidious influence of the biomedical view [36,37] has seen a “biological revolution” [38] (p. 214) or “technological paradigm” [3] dominate developments in understanding mental illness. 

Neo-liberalism assigns individual responsibility for good mental health, promoting stress management, wise eating and exercise, as individuals effectively “govern themselves” and families assume responsibility for social care. Consistent with this view, people who access mental health services are “consumers”, even if the implied choice in the selection of support is questionable. Services aim to manage demand for expensive inpatient care using assertive outreach, crisis support, home treatment and early intervention in mental health care [39]. 

“Recovery” and “risk” are integral to current mental health care in the UK [40] and Australia [41]. Moth and McKeown [35] argue that the focus of clinical practice on symptom alleviation and restoration of functioning can be seen to serve a broader agenda of mobilising a “reserve” labour force. Thus, the “return to work” and general goal setting expectations of the recovery agenda fits the neo-liberal paradigm.

#### 3.1.1. Focus of the Work

Within this dominant ideological frame, person centred, strengths based, recovery oriented, rights-based approaches to mental health social work risk being unintentionally tainted by the influence of neo-liberalism. These approaches might be seen to be consistent with mental health social work that is tasked with re-socialising individuals towards contributing and consuming. This approach is crisis driven and is preoccupied with “risk”. Underpinned by the use of psychopharmaceuticals, practice might include improving individual functioning through brief interventions, psychoeducation and the promotion of treatment adherence. At its extreme, people who have not agreed to engage with treatment and placed on compulsory orders, may still be required to pay for medication. There is no suggestion of compensation. Additionally, active discharge planning may be used to minimise hospital stays while carers, families and support persons are also leveraged to diminish the burden on the system. Other accepted practices include income management by the state. Further, rights are exercised on the basis of an individual’s capacity to stake a claim, not offered as a recognition of the collective responsibility of citizenship. Rapid but uncoordinated development in the sector has led to an unwieldy and complex service network that social workers must negotiate [40].

#### 3.1.2. Preparing for Practice

This context requires educators to prepare future mental health social workers with knowledge and practice skills which are in competition with the social work values that educators are instilling. The focus for development is on technical skills such as assessment and triaging, referring consumers to the least expensive service type, discharge planning, family psychoeducation, motivational interviewing and asset-based community development. Graduates need to be prepared to practice in environments where they, and the service they work in, maintain a competitive edge while driving efficiencies, and build evidence of the value of their contribution to the economy. Thus, workplaces are becoming “frequently toxic for mental health” [35] (p. 378). There is increasing reliance on technological interventions to drive efficiencies, privatisation of health care, targeting of the social work role to risk management and risk aversion and with it, enabling reduced length of stay in hospitals [42]. However, performance accountability measures detract from social work staff time and ability to meet recovery goals [43]. Dawson et al. found that market-oriented principles restricted the capacity of social workers to transform services and lowered staff morale [43]. 

### 3.2. Critical Social Work in Mental Health

Critical social work is consistent with the emancipatory values and holistic thinking underpinning social work practice [44,45,46]. In contrast to a biomedical paradigm, critical approaches view mental health from the perspective of power and oppression. This approach highlights dualities like the patient/carer or mentally well/ unwell and the dialectical relationships in which they exist, where one is defined in opposition to the other. Labels of pathology, diagnosed by the “powerful”, are criticised for overlooking or ignoring the social origins of distress or trauma. Recovery firstly requires the recognition of personalised pathology as internalised oppression [47]. Banning together with others of similarly oppressive experiences supports depersonalisation and breaking free from imposed views. Rather than promoting the market economy, this view expects the state to redistribute power, or more specifically wealth, and deliver services. In brief:

… critical social work practice is primarily concerned with practising in ways which further a society without domination, exploitation and oppression. It will focus both on how structures dominate, but also on how people construct and are constructed by changing social structures and relations [46] (p. 53).

#### 3.2.1. Focus of the Work

Since before the early 2000s social workers have been proposing a critical approach in mental health with little impact on the dominant paradigm [19,21,48,49]. A critical approach to mental health social work focuses on recognising and addressing power differentials. The social work role is one of co-collaborator rather than expert [50] where assessment acknowledges and undermines influences of oppression and marginalisation, challenging the dominant paradigm rather than reinforcing diagnostic categories. The Power Threat Meaning Framework, which aligns with this approach, facilitates the person to articulate their story around key themes and develop their own formulation [51]. Advocating for resources while working in allyship and arguing for the recognition of the voices of people who are disadvantaged is central to this approach.

In highlighting interpersonal power differentials, a critical approach addresses the perpetuation of relationships of power within social structures. In addition to recognising the domination of the “biomedical industrial complex” [52], critical mental health social work encourages people with similar experiences to work together such as through linking to “hearing voices” networks [53,54]. Beyond this, within organisations social work advocates for the development of policies and procedures that address questions of power.

“Mental health services rely substantially on coercive power, especially around involuntary treatment, with pervasive attention to the management of “risk” [55] (p. 123). This type of coercion and control have been the subject of critique [56] and are evidence of the oppressive practices that critical mental health social work seeks to address. 

#### 3.2.2. Preparing for Practice

Critical reflection is a core skill for practising effectively from this perspective. Reflexive practices ensure practitioners recognise the ways in which their presence and practice are manifestations of power relations and compensate for the power which is inherent in their position [27,50,57]. Essentially though “for reflection to be critical it must have as its explicit focus the uncovering and challenging of power dynamics that frame our decisions and actions” [58] (p.13). 

### 3.3. Human Rights and Social Justice

A human rights approach demands societies evolve, changing policies and practices, to recognise and embrace the diversity of their citizenship [59]. From this perspective:

good mental health and wellbeing cannot be defined by the absence of a mental health condition, but must be defined instead by the social, psychosocial, political, economic and physical environment that enables individuals and populations to live a life of dignity, with full enjoyment of their rights and in the equitable pursuit of their potential [60] (p. 1).

This approach calls for procedural and structural changes that promote global wellbeing.

#### 3.3.1. Focus of the Work

Supported by the United Nations Convention on the Rights of Persons with Disability (UNCRPD), a human rights-based approach to mental health social work recognises people as rights holders. In brief:

… the vision of the CRPD is one of engagement with actors and service providers well beyond mental health. The movement, in collaboration with the cross-disability movements worldwide, is using the CRPD to decolonise, de-economise and depsychiatrise … lives by creating excellent opportunities for participation in integrated communities. A vision for inclusion, rather than “good treatment” has emerged [61] (p. 6).

Thus, this paradigm advocates for and contributes to the development of social arrangements that promote the wellbeing of all people. From this perspective mental health social work addresses both the “upstream” context, reducing experience of coercion and marginalisation and the harm associated with coercive practice and compulsory treatment, as well as the challenges “of poverty, disadvantage and oppression affecting many people experiencing mental health difficulties” [62] (p. 885) [63]. Akin to a critical tradition, in being aware of the harms associated with coercive practices, this approach advocates alternatives for the individual and at a systemic level [56]. The contributions to changes in services and practices move beyond reinforcing dualities towards a broader social vision. These might include freedom from restrictive interventions and inhumane treatment, a commitment to non-coercive practices, respect for legal capacity and supporting economic participation and community inclusion.

#### 3.3.2. Preparing for Practice

In addition to an awareness of power and how it is exercised and mitigated, in keeping with critical perspectives, preparation for practice embodying a human rights perspective involves a broader vision of citizenship and social inclusion. Collaborating with consumer activists and advocates in allyship and co-production, and recognising the impacts of social determinants on mental health, is crucial in this work (40). Advocacy and social action target the social change that a human rights-based approach requires. As such, this approach relies on well-developed theoretical understanding and the ability to identify practical alternatives to coercive practice and involuntary treatment [56,63]. Skills in supported decision-making require an understanding of the available legal mechanisms, such as advanced statements and working alongside legal advocates [61,64]. Students must recognise the harms caused by coercive practice, embrace the concept of “dignity of risk” and have skills to work with perceived and actual risk. This requires attention to their own practice but also awareness of the context in which they work and how to support more recovery-oriented services and support. They need to hold an expectation of leadership and management that supports and enables this practice [63,64] 

### 3.4. Post-Structuralism and Social Work in Mental Health

In Baudrillard’s “Procession of the simulacrum,” the cartographer is confronted by a map of the territory which is so lifelike that he cannot distinguish the original from the copy. Conundrums that mental health social work students currently face mirror this [65]. In the age of fake news, Insta-fame and Botox, Baudrillard’s parable in many ways predicts the unreality of teaching and conducting social work in contemporary times [66,67,68]. Post-structuralism is a transformative paradigm that disconnects us from traditional notions of the real and representation, and in doing so, challenges and provides an opportunity to subvert traditional dichotomies such as those concerned with gender (man/woman) [69,70] and mental health (sane/insane) [71]. Post-structuralism frees us from the shared moral agenda which bound earlier communities together and provides a space that is amoral and individualistic [72]. Superficially, the individualism resulting from post-structuralism and neo-liberalism may appear akin. However, a deeper investigation reveals that post-structuralism is charged with cycles of creation and recreation in a neutral moral landscape while neo-liberalism is deeply bound to moral judgments and binary constructions over what is “mine”. How do we prepare social work students to practice in a mental health landscape in which deconstruction can be a liberator, or land you in the desert of the real?

#### 3.4.1. Focus of the Work

Post-structuralism loosens the ropes around who has the right and ability to define. When combined with the increased opportunity to deconstruct and create oneself anew, the boundaries around gender, sexual orientation and diversity have become more open to redefinition. For example, the emergence of Pride festivals was originally a backlash from the LGBTIQ+ community and their allies against the impact of the AIDS epidemic, which brought to the forefront the stigma and marginalisation of, particularly, gay men [73]. This legacy lives on in the “Yes vote” for marriage equality and the increased emergence and visibility of the trans rights movement [74].

The right to define and infinitely redefine oneself is integral to how post-structuralism has manifested in Western culture. An innovation, such as the Rainbow Door Programme, which will receive funding under the Royal Commission into Victoria’s Mental Health System, responds to continued prejudice and the increased prevalence of poor mental health outcomes in the LGBTIQ+ community [75]. Therefore, it is essential that acceptance of diversity is reflected in the education of mental health social workers and fostered through critical reflection [76].

#### 3.4.2. Preparing for Practice 

Social work is a discipline that is innately bound to working with structures and systems in order to support often marginalised people. In this context, it can be difficult to grapple with the tension between the structural elements inherent to social work and the anarchy of post-structuralism. For example, although avenues to construct and state our preferences, as in the case of Advanced Statements, which outline a person’s preferences regarding compulsory mental health treatment, are more available, they can be disregarded at the whim of the system. In this sense it is not sufficient to teach emerging social workers that we can merely construct ourselves, as we are still subject to the limitations of the systems which govern us and the reality that many people are still subject to prejudice and discrimination.

## 4. Discussion

By exploring the influences of a range of paradigms on mental health social work, this paper reveals tensions in practising in complex mental health systems where competing perspectives are emerging to complement, or perhaps supersede, the dominant biomedical view. Regardless of the setting, mental health social work practice embraces allyship and co-production. It is person centred, strengths based, family inclusive, trauma informed and recovery oriented [9,10,11,12]. As the profession’s mandate is of a critical hue, it aligns well with both critiques of the limitation of the current paradigmatic influences on mental health as well as proposed solutions [1,2].

While the above approaches are core to mental health social work practice, the ways in which they are conceptualised and performed are influenced by the paradigmatic view of the practitioner and the services in which they work. By way of example regarding family inclusive practice, a neo-liberal perspective might assume, and hence impose, family caring responsibilities [77,78]. Alternatively, a critical approach to family inclusive practice would welcome advocacy for families and assert their right to be supported, including fiscally [79]. A human rights approach would privilege the perspective of the person over their family and not necessarily see the family as deserving support in their own right [79]. A post-structural view would challenge prescriptions of “family” [79]. As another example from a neo-liberal perspective, person centred approaches “empower” the person by educating them about symptomatology and treatment adherence. In contrast, a critical view recognises the disempowerment of conscription into the role of patient and connects with the person beyond symptoms and their management. A human rights view would recognise the intersections between multiple avenues of disadvantage that might impact the person’s experience and mobilise resources to address them with the fundamental goals of promoting self-determination and safeguarding autonomy. Finally, a post-structural view would privilege the person’s unique understanding of their experience as they construct and reconstruct it. Understanding these influences supports graduates to locate their practice and, using critical reflection, consider the implications of their approach [24,30].

Descriptions by and for existing practitioners, and emerging practitioners, in recent years have generally been oriented towards preparing students and colleagues for the realities of practice within a setting with an established medical hierarchy, and further where the largest single discipline workforce by far is nursing. Mental health social work texts have pragmatically needed to focus upon medical terminology, mental state examination, needs assessment and intervention, legislation and provisions for compulsory treatment, and working within multi-disciplinary team structures [9,18]. A groundswell of renewed thinking about differential ideas about “evidence”, whose evidence informs good practice, service user choice and the potential of rights-based approaches, have reopened the possibilities for social work to contribute to transformative approaches that are respectful, inclusive, individuated, collaborative, holistic and anti-oppressive; that is a values-based lens towards practice [16,19,54,80]. Conversely, the recognition of the intent of practice is sometimes allusive, and potentially “invisible social work” [81]. Radical practices may be quietly “smuggled in” and subversive [13] while appearing to comply with the dominant approach. Such subtlety might be strategic in achieving outcomes in complex systems, but these potentially transformative practices risk being unrecognised. Sadly, they may be mistaken for being complicit.

Perspective transformation begins in the classroom, and requires reinforcement in field education [25,82], support through effective supervision of emerging practitioners and communities of practice. Working within neo-liberal contexts restricts progress towards change, demanding conformity to dominant practices. By encouraging future practitioners to engage in reflection that is critical, recognising the influence of assumptions prescribed by paradigms, we are preparing graduates to think critically and ensure their espoused approach is recognised in their practice. Negotiating across points of difference is complex. We anticipate that these future practitioners will be confident and capable of working within the context of dominant paradigms. They will be able to identify points of synergy and points of divergence. They will also make informed choices in their practice. Finally, they will recognise the complexity of eclecticism rather than adopting it naïvely. Tucker and Webber [40] revealed that mental health social workers “adapt their professional positioning on a fluid, contextual, basis, moving, for example, from advocate to therapist to care coordinator depending on the current demands of their role” (p. 557). As boundary spanners [80], mental health social workers position themselves on the margins and across the boundaries of services and relationships. We argue that to work effectively at such boundaries, social workers need to bring an understanding and appreciation of the worldviews that influence mental health care systems and exercise diplomacy. Thus, personal and professional responsibility is informed by multiple perspectives and underpinned by robust critical reflective pedagogy.

Incorporating a multiplicity of perspectives towards practice can be evident in research and evaluation in contemporary mental health social work, a clear example being the way Kate Day—drawing on her practice-based research—articulates social work practice in early psychosis. She notes it to be inclusive of “mitigating risk and alleviating symptoms” (p. 101) while also emphasising “self-determination” and “connectedness, hope and optimism about the future, identity, meaning in life, and empowerment” (p. 103) and focusing on “social, occupational, recreational and relationship pursuits of individuals” (p. 103) [19]. This learning intends to be critically reflective, reflexive and personally and professionally transformative [23,24,25,26]. Innovative practice in mental health may be disorienting, however, “a disorienting experience is the foundation for transformation” [24] (p. 491). Social work has a mandate for realising social justice. Recognising this mandate as a professional responsibility rather than a personal preference may reinvigorate mental health social work towards a collective position that supports courageous actions.

### Limitations

This research explored paradigms commonly regarded as framing social work practice but it is not an exhaustive coverage of potentially transformative paradigms [32,45,83]. We acknowledge the value of First Nations conceptualisations of mental health and wellbeing, recognising its absence in this paper. We look forward to learning from and with First Nations colleagues. Eco-social work also proposes a holistic perspective which we would like to explore into the future in relation to how it translates to mental health social work.

## 5. Conclusions

Global and local mental health service delivery appears to be on the cusp of major reform that is in alliance with social work theory and values [2,6,60]. Evidence of injustice is pervasive in the current mental health system. The rates of compulsory treatment remain high across the world and recent evidence suggests that people who experience area-level deprivation [84] and people from culturally and linguistically diverse backgrounds are at risk [85,86]. Drivers for change include the United Nations Human Rights Council [1], the WHO [6], the UNCRPD [87], the increased awareness and emphasis on the social determinants and role of trauma in mental distress [51] and increased preparedness to challenge the dominance of the biomedical approach [3]. Mental health social work is ideally placed to engage in this transformation. Increasing future practitioners’ awareness of the influence of paradigms on mental health practice enables the confident articulation and effective enactment of alternatives.
